# CoDC: Accurate Learning with Noisy Labels via Disagreement and Consistency

**DOI:** 10.3390/biomimetics9020092

**Published:** 2024-02-03

**Authors:** Yongfeng Dong, Jiawei Li, Zhen Wang, Wenyu Jia

**Affiliations:** 1 School of Artificial Intelligence, Hebei University of Technology, Tianjin 300401, China; dongyf@hebut.edu.cn (Y.D.); lijiawei1024@foxmail.com (J.L.); jiawenyu2021@163.com (W.J.); 2 Hebei Province Key Laboratory of Big Data Computing, Hebei University of Technology, Tianjin 300401, China; 3 Hebei Engineering Research Center of Data-Driven Industrial Intelligent, Hebei University of Technology, Tianjin 300401, China

**Keywords:** biological nervous system, DNNs, label noise, disagreement, consistency

## Abstract

Inspired by the biological nervous system, deep neural networks (DNNs) are able to achieve remarkable performance in various tasks. However, they struggle to handle label noise, which can poison the memorization effects of DNNs. Co-teaching-based methods are popular in learning with noisy labels. These methods cross-train two DNNs based on the small-loss criterion and employ a strategy using either “disagreement” or “consistency” to obtain the divergence of the two networks. However, these methods are sample-inefficient for generalization in noisy scenarios. In this paper, we propose CoDC, a novel **C**o-teaching-basedmethod for accurate learning with label noise via both **D**isagreement and **C**onsistency strategies. Specifically, CoDC maintains disagreement at the feature level and consistency at the prediction level using a balanced loss function. Additionally, a weighted cross-entropy loss is proposed based on information derived from the historical training process. Moreover, the valuable knowledge involved in “large-loss” samples is further developed and utilized by assigning pseudo-labels. Comprehensive experiments were conducted on both synthetic and real-world noise and under various noise types. CoDC achieved 72.81% accuracy on the Clothing1M dataset and 76.96% (Top1) accuracy on the WebVision1.0 dataset. These superior results demonstrate the effectiveness and robustness of learning with noisy labels.

## 1. Introduction

Inspired by the biological nervous system, deep neural networks (DNNs) have been successfully utilized in various tasks [1,2,3,4,5,6], particularly in computer vision [7,8,9,10,11,12]. This is made possible by large-scale datasets with accurate labels, although collecting them can be challenging and costly, especially in certain professional fields that require personnel with relevant professional knowledge to label samples. As a result, researchers often seek cost-effective solutions such as crowdsourcing [13], web-crawling [14], and search engine queries [15] to build datasets. However, these methods can introduce noise to the labels, which can seriously affect the generalization of DNNs during the training process [16].

Learning with noisy labels has become a popular research topic; finding ways to reduce the impact of noisy labels on networks is key to solving the problem. Existing methods include designing robust loss functions and sample selection techniques, which are often combined to create a more effective model. Co-teaching [17], a recent approach, trains two networks that each treat small-loss samples as clean and then uses them to update each other’s parameters. By starting with different random parameters, the networks are able to filter different types of errors, resulting in a more robust model. Other approaches, such as Co-teaching+ [18] and CoDis [19], have introduced disagreement strategies for sample selection and training, based on the view that the differences between the two networks are beneficial to their overall robustness. JoCoR, on the other hand, uses joint training and co-regularization to select samples with more consistent predictions and trains both networks simultaneously, arguing that disagreement strategies may not always select truly clean samples. The MLC [20] provides a detailed analysis of the significance of maintaining disagreement.

However, these methods constrain DNNs to one of a “disagreement” or “consistency” perspective, meaning that valuable knowledge involved in “high-loss” samples is ignored. Thus, we propose a new method called CoDC (Accurate Learning with Noisy Labels via Disagreement and Consistency). Specifically, we train two networks with disagreement loss at the feature level to prevent the two networks from becoming a self-training network. At the prediction level, we apply consistency loss to ensure that the predicted results of the network remain consistent. The joint application of disagreement loss and consistency loss can constrain the networks and the sample selection, enabling them to maintain a balance between disagreement and consistency and enhancing the generalization ability of the resulting network.

Furthermore, cross-entropy loss functions have been shown to exhibit an overfitting phenomenon [13] and DNNs have strong learning ability, making it inevitable that they will fit part of the noise from labels during training. By analyzing the historical training situation, we re-weight the cross-entropy loss to obtain the modified classification loss, which reduces the overfitting phenomenon of the network. Instead of discarding noisy samples directly, we use a peer network to guide learning, further improving the model’s robustness. The main contributions of this work can be summarized as follows:

- A weighted cross-entropy loss is proposed to remit the overfitting phenomenon, and the loss weights are learned directly from information derived from the historical training process.
- We propose a novel method called CoDC to alleviate the negative effect of label noise. CoDC maintains disagreement at the feature level and consistency at the prediction level using a balance loss function, which can effectively improve the generalization of networks.

## 2. Related Work

**Robust loss.** Robust loss functions have been widely explored in supervised learning to address the issue of overfitting caused by noisy labels. Several loss functions have been proposed to improve robustness against noisy labels, including Mean Absolute Error (MAE) [13], Reverse Cross-Entropy (RCE) [21], Generalized Cross-Entropy (GCE) [22], Active Passive Loss (APL) [23], Negative Learning+ (NL+), and Positive Learning+ (PL+) [24]. Ghosh et al. [13] demonstrated the effectiveness of symmetric loss in handling noisy labels and introduced the MAE loss. Wang et al. [21] proposed RCE, which performs well in multi-classification tasks but may result in underfitting. Zhang et al. [22] designed GCE by combining MAE and CE, further enhancing robustness against noisy labels and mitigating underfitting issues. Ma et al. [23] introduced the APL loss to improve the convergence of the loss function. Kim et al. [24] proposed NL+ and PL+ to address the underfitting problem of robust loss functions by analyzing the loss gradient.

**Label correction.** Label correction is a crucial technique for improving the performance of deep learning models trained on noisy datasets. Label Smoothing [25], a commonly used method, is known to act as a regularization technique that helps the model parameters converge to a small norm solution [26]. Generating pseudo-labels for noisy samples is another approach that can make full use of all samples’ information. The Meta Pseudo-Labels [27] method treats suspected noise samples in training data as unlabeled data for semi-supervised learning. PENCIL [28] uses given labels to learn from the network and updates and corrects noisy labels through the prediction results of the network, thereby improving the prediction ability of the model. However, the effectiveness of label correction methods is highly dependent on the model’s accuracy. Erroneous labels may be generated constantly, resulting in the accumulation of errors if the model’s accuracy is poor. Co-LDL [29] is a method that trains on high-loss samples using label distribution learning and enhances the learned representations using a self-supervised module, which further boosts model performance and the use of training data.

**Small-loss samples.** The existing methods mainly apply small-loss criteria for selection. They consider the small-loss samples as clean samples and the large-loss samples as noisy samples, then adopt different strategies for learning. MentorNet [30] pretrains a teacher network to select clean samples, which then guides the student network. However, this method has the disadvantage of cumulative error caused by sample selection bias. Co-teaching [17] trains two networks; each network selects small loss samples to send to the peer network for learning. However, due to continuous peer-to-peer learning, the parameters of the two networks will gradually become consistent. Therefore, Co-teaching+ [18] only chooses the samples with divergent predictions between the two networks for learning. Similarly, CoDis [19] applies the regular variance term and selects samples with large variance to send to the peer network for learning in order to avoid consistency between the two networks. However, JoCoR [31] assumes that few of the samples with divergent predictions are likely to be clean. It is recommended that the two networks be trained jointly using samples with predictive consistency. As a result, the two networks will easily converge to a consensus or even produce the same prediction error. Liu et al. [20] analyzed the existence of appropriate “differences” between the two networks in Co-teaching, which is beneficial to the improvement of model robustness. In contrast, our proposed approach takes into account both the differences and consistency of the two networks.

In addition, DivideMix [32] fits the loss of all samples into a Gaussian mixture model (GMM), based on the idea that the loss distribution of all samples will conform to two Gaussian distributions. The clean samples belong to the component with a lower central value, while the noisy samples belong to the component with a higher central value, allowing the noisy and clean samples to be divided. LongReMix [33] selects clean samples with high confidence through a two-stage selection method. Kong et al. [34] proposed a penalty label and introduced new sample selection criteria to screen clean samples. However, these methods essentially apply the small-loss sample selection principle.

## 3. Materials and Methods

### 3.1. Preliminary

In this paper, we consider a classification task with *C* classes. The dataset contains noisy labels. $D={\left\{\left(x_{i},{\tilde{y}}_{i}\right)\right\}}_{i=1}^{n}$, where *n* is the sample size, $x_{i}$ is the i-th sample, and ${\tilde{y}}_{i}\in \left[C\right]$ is the label with noise. For ${\tilde{y}}_{i}$, its true and unobservable label is represented by $y_{i}$. As in Co-teaching, two networks are trained, denoted as $f_{1}=f\left(x,\theta_{1}\right)$ and $f_{2}=f\left(x,\theta_{2}\right)$. The two networks have the same architecture and are initialized by random initialization, meaning that they have different network parameters. In this method, $p_{1}=\left[p_{1}^{1},p_{1}^{2},\ldots ,p_{1}^{C}\right]$ and $p_{2}=\left[p_{2}^{1},p_{2}^{2},\ldots ,p_{2}^{C}\right]$ represent the respective prediction probabilities of the two networks for the samples. The aim is to learn a robust classifier that can be trained on D to obtain correct predictions.

### 3.2. Training with Disagreement and Consistency

In this section, we mainly introduce the training functions used in our method, including the modified classification loss, balance loss, and peer network guiding learning.

**Modified classification loss.** During the initial training phase, Cross-Entropy (CE) is utilized as the classification loss. Considering that the two networks possess distinct learning capabilities, the cross-entropies of both networks are summed to minimize the distance between them in terms of predicting clean samples and their labels. This approach helps to minimize erroneous information. The classification loss can be represented as follows.

(1) $$\begin{array}{cc} L_{c\;}\left(x_{i},{\tilde{y}}_{i}\right) & =\ell_{\mathrm{CE}1}\left(x_{i},{\tilde{y}}_{i}\right)+\ell_{\mathrm{CE}2}\left(x_{i},{\tilde{y}}_{i}\right)\\ \; & =-\sum_{i=1}^{N}\sum_{m=1}^{C}{\tilde{y}}_{i}\log\left(p_{1}^{m}\left(x_{i}\right)\right)\\ \; & -\sum_{i=1}^{N}\sum_{m=1}^{C}{\tilde{y}}_{i}\log\left(p_{2}^{m}\left(x_{i}\right)\right) \end{array}$$

In previous research, it has been discovered that CE can overfit noise label learning [13]. The small-loss sample selection strategy can result in incorrect selection of clean samples. As the number of epochs increases, CE may cause the network to fit noise labels, which can negatively impact the model’s generalization. To address this issue, we propose a modified loss function that optimizes the weight of CE for each sample and reduces the fitting noise by combining the historical law of sample loss. Specifically, when the model separates clean and noisy samples, the classification loss of each sample should be maintained at its original value and should not fluctuate greatly during the learning process. Due to the overfitting problem, the loss of noisy samples with large losses may gradually decrease, resulting in the classifier’s failure to correctly distinguish between clean and noisy samples.

Therefore, we have modified the classification loss to stabilize the sample loss after the network separates clean and noisy samples. We define the set $L_{s}=\left\{L_{c_{t-1}},L_{c_{t-2}},\cdots ,L_{c_{t-s}}\right\}$ to record the value of the classification loss of sample *x* in each epoch of training, where *t* is the current number of training epochs and *s* is the length of the set. We define $L_{\mathrm{history}}$ as follows.

(2) $$L_{\mathrm{history}\;}=\left|\frac{1}{s}\sum_{i=t-s}^{t-1}L_{c_{i}}\right|$$

The difference between the loss of the current sample and the loss of the training history, denoted by $\left|L_{c_{t}}-L_{\mathrm{history}\;}\right|$, can indicate the model’s training stability. To ensure stable training and prevent the model from overfitting on noisy samples, we utilized the modified classification loss $L_{\mathrm{mcl}}$ to train the model. Minimizing $L_{\mathrm{mcl}}$ enhances the stability of the training process.

(3) $$L_{\mathrm{mcl}}=\left|L_{c_{t}}-L_{\mathrm{history}\;}\right|$$

**Balance loss.** Co-teaching [17] has been successful in the field of learning with noisy labels. The approach involves training two networks simultaneously, with each network feeding what it deems to be clean samples to its peer network for learning. Because the initial random parameters of the two networks are different, they can filter different types of errors, which improves the generalization ability of the networks. However, as the two networks learn from each other their parameters gradually converge, resulting in decreased ability to filter different errors [18,20].

To control the disagreement between the dual networks at the feature level and encourage them to learn more feature knowledge, we introduce the disagreement loss $L_{\mathrm{dis}}$. The cosine similarity is used to calculate the output features of the two networks in order to measure such feature differences. The equation is as follows:

(4) $$\begin{array}{cc} L_{\mathrm{dis}}\; & =CosineSimilarity(z_{1}\left(x_{i}\right),z_{2}\left(x_{i}\right))\\ \; & =\sum_{i=1}^{N}\sum_{m=1}^{K}\frac{z_{1}^{m}\left(x_{i}\right)\cdot z_{2}^{m}\left(x_{i}\right)}{∥z_{1}^{m}\left(x_{i}\right)∥\times ∥z_{2}^{m}\left(x_{i}\right)∥} \end{array}$$

where $z_{1}$ and $z_{2}$ are the feature representations of the sample in network one and network two, respectively, and *K* is the dimension of the feature. We apply $L_{\mathrm{dis}}$ in the final loss function to control the difference between the two networks, encourage the networks to learn more at the feature level, and inhibit the convergence of the two networks.

In combination with the theory summarized above, we encourage the two networks to maintain differentiation at the feature level, preventing the two networks from reaching a consensus to ensure that more knowledge is learned. However, from the view of agreement maximization principles [35], different networks will agree on the prediction of clean samples. Thus, although the differentiation in features is encouraged, the two networks should be consistent in the prediction of clean samples. We designed the consistency loss $L_{\mathrm{con}}$ to minimize the two networks’ predictions $p_{1}$ and $p_{2}$. Specifically, we apply the Jensen–Shannon (*JS*) Divergence, which measures the difference between two probability distributions, to capture the consistency of two networks’ predictions:

(5) $$\begin{array}{cc} L_{\mathrm{con}}\; & =D_{JS}\left(p_{1}∥p_{2}\right)\\ \; & =\frac{1}{2}D_{KL}\left(p_{1}∥\frac{p_{1}+p_{2}}{2}\right)+\frac{1}{2}D_{KL}\left(p_{2}∥\frac{p_{1}+p_{2}}{2}\right) \end{array}$$

where

$$D_{KL}\left(p_{1}∥\frac{p_{1}+p_{2}}{2}\right)=\sum_{i=1}^{N}\sum_{m=1}^{C}p_{1}^{m}\left(x_{i}\right)\log\frac{2p_{1}^{m}\left(x_{i}\right)}{p_{1}^{m}\left(x_{i}\right)+p_{2}^{m}\left(x_{i}\right)}$$

$$D_{KL}\left(p_{2}∥\frac{p_{1}+p_{2}}{2}\right)=\sum_{i=1}^{N}\sum_{m=1}^{C}p_{2}^{m}\left(x_{i}\right)\log\frac{2p_{2}^{m}\left(x_{i}\right)}{p_{1}^{m}\left(x_{i}\right)+p_{2}^{m}\left(x_{i}\right)}.$$

We weight the $L_{\mathrm{dis}}$ and $L_{\mathrm{con}}$ to obtain $L_{\mathrm{balance}}$.

(6) $$L_{\mathrm{balance}}=\beta L_{\mathrm{dis}}+\gamma L_{\mathrm{con}}$$

The significance of Equation (6) is that we aim for the two networks to maintain differences at the feature level while making the same predictions.

**Peer network guided learning.** Many previous works have simply discarded noisy samples, resulting in insufficient training due to the decrease in training samples. Semi-supervised learning strategies can be used to generate pseudo-labels to replace the given labels for robust learning. However, the network’s generalization ability affects the quality of the pseudo-labels. Moreover, the network tends to fall into self-cognitive errors as the number of network iterations increases, causing the self-generated pseudo-labels to accumulate errors. Therefore, we used a peer network to guide learning. The peer network prediction *p* is used for pseudo-label learning, and the cross-entropy loss can be formulated as follows:

$$\ell_{pl1}=\ell_{\mathrm{CE}}\left(p_{1}\left(x_{i}\right),\varepsilon \left(p_{2}\left(x_{i}\right),\tau \right)\right),$$

$$\ell_{pl2}=\ell_{\mathrm{CE}}\left(p_{2}\left(x_{i}\right),\varepsilon \left(p_{1}\left(x_{i}\right),\tau \right)\right),$$

where

(7) $$\varepsilon \left(p,T\right)=\frac{\exp(p/T)}{\sum_{m=1}^{C}\exp\left(p^{m}\left(x_{i}\right)/T\right)}.$$

The sharpening function ε(p,T) is utilized to enhance the guidance capabilities of the peer network, where *T* is the temperature parameter. To align with Jo-SRC [36], we set τ = 0.1. However, there may be label errors due to the network’s limited generalization ability. To prevent the network from fitting to these errors, a small weight λ is applied; in our experiments, λ was typically set to 0.5. Additionally, we employed weak data augmentation techniques such as random cropping and random symmetry flipping to further enhance the guidance capabilities of the peer network. Peer-to-peer guided learning is described as follows.

(8) $$L_{pl}=\lambda \left(\ell_{pl1}+\ell_{pl2}\right)$$

**Training loss.** Finally, the training loss is constructed as follows.

(9) $$L=\alpha L_{\mathrm{mcl}}+L_{\mathrm{balance}}+L_{pl}$$

Unlike Co-teaching, Equation (9) is used to train the two networks simultaneously.

Small-loss samples are more likely to be clean samples; therefore, we are more inclined to select these as clean samples for learning. As mentioned above, we aim to add more samples that exhibit different features in the two networks while maintaining consistent prediction results for learning. Such samples are also considered cleaner. Therefore, we define the loss of sample selection in the training process as follows.

(10) $$L_{\mathrm{select}}=\alpha L_{\mathrm{mcl}}+L_{\mathrm{balance}}$$

As in Equation (6), the $L_{\mathrm{balance}}$ is used to ensure a balance between feature differences and prediction consistency, with α controlling the weight of the modified classification loss.

Equation (10) is used to select clean samples for learning, while the remaining samples are considered to be noise samples. The noise samples are learned using the peer network guided learning method to enhance the network’s robustness.

Considering the ability of deep networks to learn clean samples first, we warmed up the network at the beginning of training using all samples and employed the cross-entropy loss as the training loss. After the warm-up period, we selected the top 1−τ small-loss samples as clean samples and considered the rest to be noise samples, where τ represents the noise rate in the dataset. Specifically, our approach is as follows:

(11) $${\tilde{D}}_{n}=\arg\;\min_{D_{n}^{\prime }:\left|D_{n}^{\prime }\right|\geq \left(1-\tau \right)\left|D_{n}\right|}L_{\mathrm{select}}\left(D_{n}^{\prime }\right).$$

**Figure 1:**
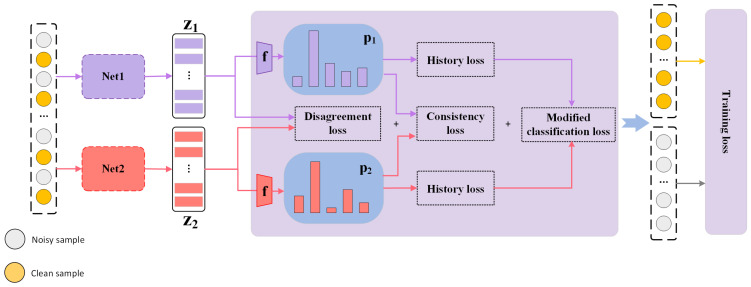
Sample selection of CoDC. For each sample, the classification loss, disagreement loss, and consistency loss are calculated and the small-loss samples are taken as the clean sample. In general, the essence of this method is to maintain a balance of disagreement and consistency between the two networks to achieve the best performance.

Our sample selection method is depicted in Figure 1. The pseudocode is presented in Algorithm 1.
**Algorithm 1** CoDC algorithm**Input**: two networks with weights $\theta_{1}$ and $\theta_{2}$, learning rate η, noise rate τ, epoch $T_{warmup}$ and $T_{max}$, iteration $t_{max}$;
  1:**for** $T=1\to T_{max}$ **do**  2:   **Shuffle** training dataset D;  3:   **if** $T<T_{warmup}$ **then**  4:     training by Cross-Entropy;  5:   **else**  6:     **for** $t=1\to t_{max}$ **do**  7:        **Fetch** mini-batch $D_{n}$ from *D*;  8:        **Obtain** ${\tilde{D}}_{n}$ by $L_{\mathrm{select}}$;  9:        **Update** $\left(\theta_{1},\theta_{2}\right)=\left(\theta_{1},\theta_{2}\right)-\eta L\left({\tilde{D}}_{n}\right)$;10:     **end for**11:   **end if**12:**end for**
**Output**: $\theta_{1}$ and $\theta_{2}$


## 4. Results

### 4.1. Datasets and Implementation Details

**Datasets and noise types.** We used four benchmark datasets with noise (F-MNIST [37] (http://fashion-mnist.s3-website.eu-central-1.amazonaws.com/, accessed on 12 November 2023), SVHN [38] (http://ufldl.stanford.edu/housenumbers/, accessed on 12 November 2023), CIFAR-10 [39] (https://www.cs.toronto.edu/~kriz/cifar-10-python.tar.gz, accessed on 12 November 2023), and CIFAR100 [39] (https://www.cs.toronto.edu/~kriz/cifar-100-python.tar.gz, accessed on 12 November 2023)) and two real-world datasets (Clothing1M [40] (https://opendatalab.com/OpenDataLab/Clothing1M, accessed on 12 November 2023) and WebVision1.0 [41] (https://data.vision.ee.ethz.ch/cvl/webvision/dataset2017.html, accessed on 12 November 2023)) to evaluate our method. F-MNIST (Fashion-MNIST) contains ten classes of grayscale images, each sized 28 × 28, with 60,000 training images and 10,000 test images. SVHN is derived from Google Street View House Number, including ten classes of color images, each sized 32 × 32, with 73,257 training images and 26,032 test images. Both the CIFAR-10 and CIFAR-100 datasets contain 60,000 training images and 10,000 test images, each sized 32 × 32. CIFAR-10 comprises ten classes, while CIFAR-100 comprises 100 classes.

Clothing1M is made up of one million training images collected from online shopping sites and contains fourteen classes with labels generated using surrounding text. WebVision1.0 is a large dataset with real-world noise labels. It has taken over 2.4 million images from the internet, including 1000 classes contained in ImageNet ILSVRC2012 [42]. In order to facilitate comparison with other methods, we followed the previous work and obtained the first 50 classes of images from Google images for both training and testing.

For SVHN datasets, the number of samples in each class is unbalanced. Specifically, the number of samples in each class is 13,861, 10,585, 8497, 7458, 6882, 5727, 5595, 5045, 4659, and 4948 respectively. For F-MNIST, CIFAR-10, CIFAR-100, Clothing1M, and WebVision1.0 datasets, the distribution of samples in each class is uniform.

Clothing1M and WebVision1.0 are real-world datasets that contain noisy labels. All of the benchmark datasets are clean; thus, we set up the label transition matrix [25] and manually added noise to test the validity of the method. Our experiment mainly followed [43], and we conducted experiments with a variety of noise types: symmetric noise, pairflip noise, tridiagonal noise, and asymmetric noise. Noise rates of 20% and 40% were tested for each type of noise. It is necessary to ensure that the number of clean samples is greater than the number of noisy samples; otherwise, it will be impossible to distinguish the real class of samples [23]. The construction of each noise type was as follows:

(1) Symmetric noise: the clean labels in each class are uniformly flipped to labels of other wrong classes.
(2) Asymmetric noise: considers the visual similarity in the flipping process, which is closer to real-world noise; for example, the labels of cats and dogs will be reversed, and the labels of planes and birds will be reversed. Asymmetric noise is an unbalanced type of noise.
(3) Pairflip noise: this is realized by flipping clean labels in each class to adjacent classes.
(4) Tridiagonal noise: realized by two consecutive pairflips of two classes in opposite directions.

**Figure 2:**
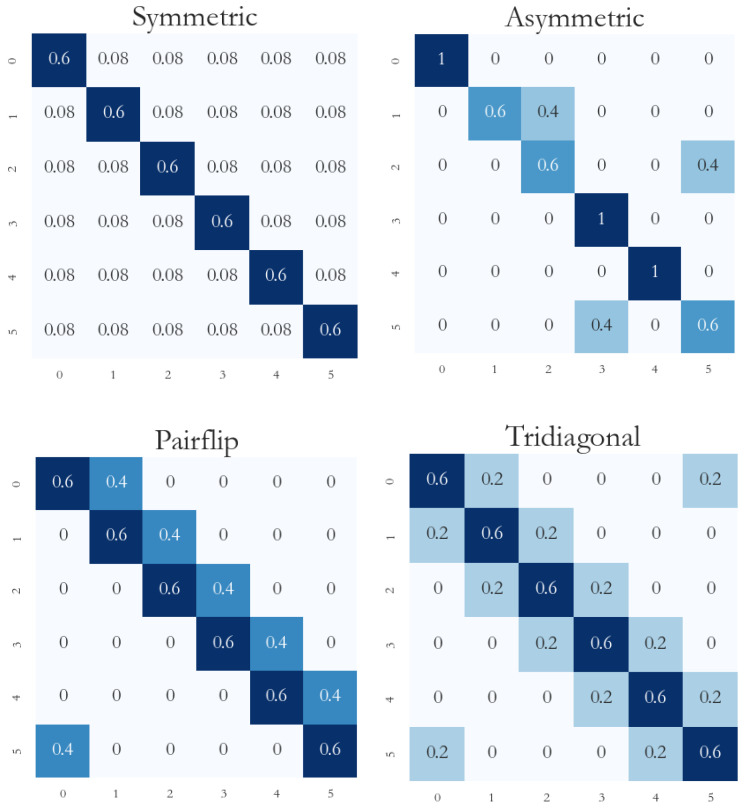
Label transition matrix.

The label transition matrix is shown in Figure 2, taking six categories and a 40% noise rate as an example.

**Figure 3:**
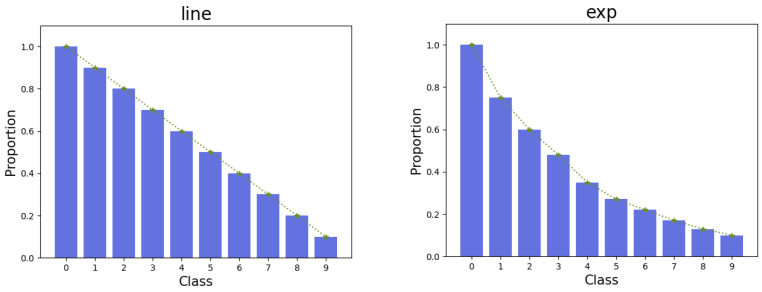
Construction of two long-tailed distributed datasets.

In addition, we conducted experiments on noisy long-tailed datasets [19]. We first built a long-tailed distribution dataset. Specifically, we reduced the number of samples in different classes such that the dataset formed a long-tailed distribution with class imbalance [44]. In this paper, we use two ways of simulating the long-tailed distribution: exponential simulation (exp) and linear simulation (line), as shown in Figure 3. Taking SVHN as an example, the two different simulation appraoches are named SVHN-EXP and SVHN-line, respectively. We further added asymmetric noise to the long-tailed dataset and tested four different noise intensities.

**Models and parameters.** Following previous works [31], we used a nine-layer CNN for F-MNIST, SVHN, CIFAR-10, and CIFAR-100. We used a SGD optimizer with momentum set at 0.9, an initial learning rate of 0.02, and a weight decay of 0.0005. The learning rate was reduced by a factor of 10 at the 100th and 150th epochs. The batch size was set to 128, and the total number of training epochs was 200.

In the real-world datasets, for the Clothing1M dataset the training used ResNet-18 [45] pretrained on ImageNet. For WebVision, the training used inception-resnet v2 [46]. We trained the network for 100 epochs using a SGD optimizer with an initial learning rate of 0.002. The learning rate was reduced by a factor of 10 at the 30th and 60th epochs. The batch size was set to 64; the other settings were the same as those for the benchmark datasets.

We implemented all the methods with default parameters using PyTorch 1.13.1 and conducted all the experiments on a NVIDIA RTX3090 GPU.

### 4.2. Comparison with SOTA Methods

We compared our method with the Standard CE baseline and more recent SOTA methods, including:

(1) Standard: trains a single network and uses only standard cross-entropy loss.
(2) Co-teaching [17]: trains two networks simultaneously; the two networks guide each other during learning.
(3) Co-teaching-plus [18]: trains two networks simultaneously while considering the small loss samples of the two networks’ diverging samples.
(4) JoCoR [31]: trains two networks simultaneously and applies the co-regularization method to maximize the consistency between the two networks.
(5) Co-learning [47]: a simple and effective method for learning with noisy labels; it combines supervised and self-supervised learning to regularize the network and improve generalization performance.
(6) Co-LDL [29]: an end-to-end framework proposed to train high-loss samples using label distribution learning and enhance the learned representations by a self-supervised module, further boosting model performance and the use of training data.
(7) CoDis [19]: trains two networks simultaneously and applies the covariance regularization method to maintain the divergence between the two networks.
(8) Bare [48]: proposes an adaptive sample selection strategy to provide robustness against label noise.

**Results on benchmark datasets.** Table 1 shows the experimental results under four benchmark datasets with four noise types and two noise rates. The test accuracy consists of the mean and standard deviation (%) calculated from the last ten epochs of training. Based on the experimental results, we can conclude that our method is superior to state-of-the-art methods on the benchmark datasets. For example, on the CIFAR-10 dataset with symmetric noise and a 40% noise rate, our method scores 1.26% higher than Co-LDL and 10.75% higher than JoCoR. For pairflip noise with a 40% noise rate, our method scores 4.43% higher than Co-LDL and 21.17% higher than JoCoR. Because JoCoR only considers the consistency of the two networks, it is extremely easy for errors to accumulate with asymmetric and pairflip noise, which ultimately affects the generalization of the networks. This experiment proves the superiority of our method on the benchmark datasets.

**Figure 4:**
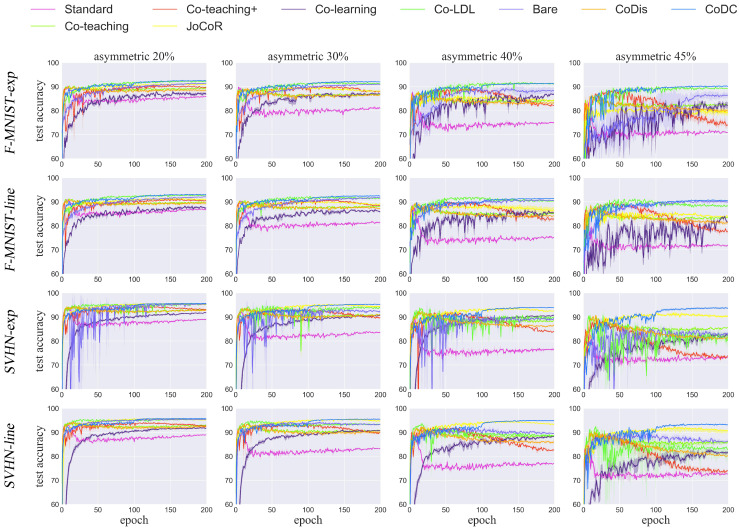
Test accuracy comparison for the number of epochs on the four long-tailed noisy datasets. Each experiment was repeated three times. The error bar for the standard deviation in each figure has been shaded.

**Results on noisy long-tailed datasets.** We tested the model on noisy long-tailed datasets with four noise rates. It can be seen that our method achieves the best performance in most cases. Although the accuracy is not higher than all advanced methods, our method remains competitive. Table 2 shows the results on the noisy long-tailed datasets. Figure 4 shows the test accuracy comparison for the number of epochs.

For the results shown in Figure 4, each experiment was repeated three times; the curve represents the average accuracy of the three experiments, while the shaded part represents the error bar for the standard deviation. From the curve, it can be seen that our method has excellent performance under both high and low noise rates. The small area of the shaded part indicates that our method has good statistical significance and is less affected by random factors.

**Results on real-world datasets.** Table 3 and Table 4 show the results on the real-world datasets Clothing1M and WebVision. In addition, we evaluated the generalization ability of these methods on ImageNet ILSVRC12. Our method achieves the best performance on real-world datasets among the compared methods.

### 4.3. Ablation Study

We conducted ablation experiments on the CIFAR-100 dataset under symmetric noise at a 40% noise rate to emphasize our design featuring the modified classification loss, balance loss, and peer network guiding learning. The accuracy when the networks lack a sample selection support strategy is only 33.20%, which is due to the influence of the noisy label environment. When the sample selection strategy is added, the accuracy increase to 64.74%. The modified classification loss, which can alleviate overfitting caused by CE, improves the accuracy by 1.17%. When the balance loss is added, the accuracy improves by 2.47%, which confirms our assumption that maintaining a balance between disagreement and consistency in the two networks can improve their learning performance. Finally, the addition of peer network guided learning further improves the accuracy of the networks. The experimental results are shown in Table 5.

### 4.4. Comparison of Running Time

We compared the running time on the CIFAR-10 dataset under symmetric noise at a 40% noise rate. All the methods were trained for 200 epochs in the same experimental environment. The results are shown in Table 6. Our method outperforms all the compared methods, with less time consumption than Co-LDL and Bare and similar time consumption to Co-teaching and Co-teaching-plus.

## 5. Conclusions

The existing co-teaching methods train two networks simultaneously; however, as iteration progresses, the parameters of the two networks gradually converge and they degenerate into a self-training network. To address this problem, we propose using the disagreement loss and consistency loss to balance the relationship between the two networks. Our ablation experiments confirm that balancing the relationship between the two networks can improve the generalization ability of the resulting model. In addition, to alleviate the over-fitting phenomenon when using cross-entropy, we propose a modified classification loss based on analyzing the change in the historical classification loss. Finally, we use a peer network to guide the learning of noisy samples to ensure that all samples are utilized for training. A large number of experiments demonstrate the effectiveness of the proposed method, providing a new research direction for co-teaching. In our future work, we intend to further explore the relationship between the two networks based on the idea of co-teaching in order to enhance their performance when learning with noisy labels; for example, more reasonable methods could be employed to balance the disagreement and consistency between the two networks, and more accurate pseudo-label correction strategies could be utilized.

## Figures and Tables

**Table 1:** Mean and standard deviations of test accuracy (%) on four benchmark datasets with four noise types and two noise rates (20%, 40%) for each noise type. The test accuracy is calculated using the last ten epochs. The best mean results are in bold.

	Noise Type	Sym		Asym		Pair		Trid	
	**Setting**	**20%**	**40%**	**20%**	**40%**	**20%**	**40%**	**20%**	**40%**
	Standard	87.46 ± 0.09	72.46 ± 0.68	89.34 ± 0.15	77.73 ± 0.19	84.23 ± 0.23	61.46 ± 0.32	85.86 ± 0.4	66.44 ± 0.28
	Co-teaching	91.20 ± 0.03	88.06 ± 0.06	91.29 ± 0.16	86.32 ± 0.09	89.97 ± 0.09	84.99 ± 0.11	90.54 ± 0.05	86.29 ± 0.09
	Co-teaching-plus	92.85 ± 0.03	91.42 ± 0.03	93.42 ± 0.02	89.38 ± 0.17	92.90 ± 0.03	88.38 ± 0.16	92.75 ± 0.08	90.40 ± 0.04
	JoCoR	94.11 ± 0.02	93.24 ± 0.01	94.24 ± 0.02	91.18 ± 0.16	93.90 ± 0.01	91.37 ± 0.21	94.03 ± 0.01	92.93 ± 0.01
F-MNIST	Co-learning	90.03 ± 0.29	90.03 ± 0.21	90.88 ± 0.22	88.67 ± 0.48	91.08 ± 0.18	87.59 ± 0.35	90.98 ± 0.16	90.27 ± 0.24
	Co-LDL	94.68 ± 0.10	93.97 ± 0.14	**95.02 ± 0.13**	**93.71 ± 0.57**	**94.84 ± 0.08**	93.66 ± 0.11	94.88 ± 0.11	**94.26 ± 0.09**
	CoDis	90.97 ± 0.05	87.92 ± 0.10	91.55 ± 0.08	85.77 ± 0.35	90.23 ± 0.04	83.92 ± 0.08	90.33 ± 0.02	86.10 ± 0.09
	Bare	93.59 ± 0.16	92.79 ± 0.13	93.79 ± 0.12	93.47 ± 0.10	93.60 ± 0.14	92.75 ± 0.13	93.64 ± 0.11	93.02 ± 0.14
	CoDC	**94.87 ± 0.06**	**94.05 ± 0.08**	94.66 ± 0.08	93.63 ± 0.04	94.64 ± 0.09	**93.67 ± 0.09**	**94.89 ± 0.06**	94.09 ± 0.08
	Standard	87.21 ± 0.10	70.97 ± 0.34	89.55 ± 0.05	77.79 ± 0.21	84.84 ± 0.10	61.39 ± 0.35	86.37 ± 0.2	67.25 ± 0.44
	Co-teaching	91.98 ± 0.04	89.21 ± 0.23	91.91 ± 0.35	88.00 ± 0.43	91.24 ± 0.27	85.94 ± 0.06	92.03 ± 0.04	87.94 ± 0.04
	Co-teaching-plus	94.71 ± 0.01	92.86 ± 0.03	94.37 ± 0.03	88.14 ± 0.06	94.15 ± 0.02	88.68 ± 0.14	94.74 ± 0.01	91.88 ± 0.06
	JoCoR	96.41 ± 0.02	95.70 ± 0.01	96.17 ± 0.02	93.89 ± 0.37	96.29 ± 0.01	93.88 ± 0.26	96.54 ± 0.01	95.29 ± 0.09
SVHN	Co-learning	93.18 ± 0.10	92.08 ± 0.11	92.74 ± 0.18	88.07 ± 0.43	93.39 ± 0.11	89.07 ± 0.16	93.48 ± 0.07	92.21 ± 0.08
	Co-LDL	**96.66 ± 0.05**	95.51 ± 0.33	96.20 ± 0.07	91.80 ± 0.30	**96.29 ± 0.07**	91.56 ± 0.23	96.42 ± 0.07	95.03 ± 0.06
	CoDis	91.67 ± 0.04	89.19 ± 0.08	92.10 ± 0.06	87.27 ± 0.18	91.25 ± 0.07	85.09 ± 0.11	91.83 ± 0.03	88.34 ± 0.01
	Bare	96.20 ± 0.06	94.92 ± 0.10	95.83 ± 0.12	92.44 ± 0.25	95.84 ± 0.18	91.66 ± 0.30	95.98 ± 0.07	94.46 ± 0.13
	CoDC	96.64 ± 0.03	**95.89 ± 0.03**	**96.26 ± 0.06**	**94.60 ± 0.08**	96.26 ± 0.04	**95.13 ± 0.04**	**96.48 ± 0.04**	**95.18 ± 0.05**
	Standard	75.74 ± 0.33	58.64 ± 0.81	82.32 ± 0.14	72.19 ± 0.32	76.38 ± 0.35	55.03 ± 0.27	76.26 ± 0.26	58.72 ± 0.26
	Co-teaching	82.24 ± 0.18	77.16 ± 0.10	80.76 ± 0.11	72.85 ± 0.11	82.55 ± 0.10	75.74 ± 0.14	82.50 ± 0.12	76.28 ± 0.12
	Co-teaching-plus	81.96 ± 0.12	71.49 ± 0.33	79.68 ± 0.13	70.96 ± 0.69	79.71 ± 0.14	58.39 ± 0.76	81.15 ± 0.05	64.79 ± 0.47
	JoCoR	85.23 ± 0.09	79.77 ± 0.18	85.62 ± 0.21	73.50 ± 1.25	81.75 ± 0.26	68.29 ± 0.30	82.78 ± 0.11	73.53 ± 0.28
CIFAR-10	Co-learning	88.82 ± 0.23	85.99 ± 0.22	88.58 ± 0.28	82.66 ± 0.44	89.13 ± 0.11	79.68 ± 0.27	89.47 ± 0.19	86.40 ± 0.23
	Co-LDL	91.57 ± 0.15	88.89 ± 0.26	90.89 ± 0.22	84.21 ± 0.26	91.10 ± 0.17	85.03 ± 0.15	91.12 ± 0.22	87.62 ± 0.19
	CoDis	82.36 ± 0.24	77.04 ± 0.09	84.58 ± 0.05	75.30 ± 0.32	82.53 ± 0.23	70.86 ± 0.22	82.69 ± 0.07	74.59 ± 0.05
	Bare	85.30 ± 0.61	78.90 ± 0.70	86.44 ± 0.39	81.20 ± 0.46	84.98 ± 0.57	74.53 ± 0.28	85.53 ± 0.45	77.40 ± 0.59
	CoDC	**92.35 ± 0.07**	**90.15 ± 0.13**	**91.53 ± 0.10**	**84.25 ± 0.12**	**92.03 ± 0.05**	**89.46 ± 0.15**	**92.59 ± 0.15**	**89.70 ± 0.18**
	Standard	46.10 ± 0.53	33.20 ± 0.58	46.11 ± 0.53	33.20 ± 0.58	46.01 ± 0.33	33.16 ± 0.32	46.30 ± 0.21	33.29 ± 0.31
	Co-teaching	50.87 ± 0.31	43.38 ± 0.35	48.88 ± 0.29	35.92 ± 0.34	49.57 ± 0.35	35.11 ± 0.45	50.14 ± 0.31	39.77 ± 0.38
	Co-teaching-plus	51.72 ± 0.33	44.31 ± 0.67	51.48 ± 0.28	34.20 ± 0.64	50.71 ± 0.77	34.29 ± 0.27	51.54 ± 0.29	41.36 ± 0.29
	JoCoR	51.61 ± 0.37	42.78 ± 0.26	51.21 ± 0.09	42.68 ± 0.23	51.46 ± 0.32	42.01 ± 0.21	51.58 ± 0.06	42.77 ± 0.34
CIFAR-100	Co-learning	62.43 ± 0.31	57.18 ± 0.35	63.04 ± 0.36	49.69 ± 0.31	62.53 ± 0.31	49.29 ± 0.41	63.26 ± 0.35	59.57 ± 0.43
	Co-LDL	66.60 ± 0.24	61.51 ± 0.34	66.85 ± 0.25	**59.98 ± 0.50**	66.59 ± 0.29	**58.87 ± 0.35**	66.53 ± 0.26	60.87 ± 0.30
	CoDis	50.65 ± 0.35	43.44 ± 0.27	50.14 ± 0.38	35.43 ± 0.38	50.80 ± 0.41	35.15 ± 0.29	51.50 ± 0.40	41.43 ± 0.45
	Bare	63.32 ± 0.32	55.33 ± 0.62	61.62 ± 0.26	40.88 ± 1.17	61.22 ± 0.51	40.92 ± 1.40	62.88 ± 0.28	48.82 ± 0.51
	CoDC	**72.42 ± 0.22**	**68.76 ± 0.16**	**70.94 ± 0.11**	57.37 ± 0.33	**70.78 ± 0.27**	56.14 ± 0.19	**70.78 ± 0.17**	**61.67 ± 0.16**

**Table 2:** Mean and standard deviations of test accuracy (%) on four noisy long-tailed datasets with four different noise rates (20%, 30%, 40%, 45%). The test accuracy is calculated using the last ten epochs. The best mean results are in bold.

	Noise Type	Asym.20%	Asym.30%	Asym.40%	Asym.45%
	Standard	85.77 ± 0.17	80.99 ± 0.45	74.90 ± 0.25	70.90 ± 0.37
	Co-teaching	89.41 ± 0.13	86.83 ± 0.16	84.21 ± 0.12	82.27 ± 0.21
	Co-teaching-plus	90.00 ± 0.17	88.60 ± 0.20	82.36 ± 0.26	76.19 ± 0.57
	JoCoR	91.00 ± 0.10	88.12 ± 0.12	84.10 ± 0.17	78.41 ± 0.29
F-MNIST-exp	Co-learning	86.70 ± 0.25	86.95 ± 0.29	86.78 ± 0.41	83.02 ± 0.66
	Co-LDL	92.16 ± 0.11	91.40 ± 0.16	91.28 ± 0.21	89.31 ± 0.20
	CoDis	89.11 ± 0.17	87.11 ± 0.16	83.84 ± 0.26	80.88 ± 0.13
	Bare	91.43 ± 0.15	90.89 ± 0.18	88.45 ± 1.31	86.41 ± 1.15
	CoDC	**92.45 ± 0.08**	**92.24 ± 0.10**	**91.37 ± 0.06**	**90.20 ± 0.11**
	Standard	86.94 ± 0.22	81.38 ± 0.33	75.26 ± 0.39	71.68 ± 0.32
	Co-teaching	89.79 ± 0.10	87.89 ± 0.15	85.24 ± 0.26	83.43 ± 0.14
	Co-teaching-plus	90.61 ± 0.14	87.79 ± 0.20	82.83 ± 0.35	77.28 ± 0.58
	JoCoR	91.12 ± 0.15	89.02 ± 0.17	85.29 ± 0.26	84.01 ± 0.26
F-MNIST-line	Co-learning	87.84 ± 0.23	86.22 ± 0.41	85.66 ± 0.43	83.09 ± 1.04
	Co-LDL	92.73 ± 0.21	91.47 ± 0.24	89.98 ± 0.38	88.39 ± 0.47
	CoDis	90.13 ± 0.10	87.79 ± 0.16	84.77 ± 0.26	81.46 ± 0.13
	Bare	92.11 ± 0.13	91.79 ± 0.09	90.55 ± 0.28	89.92 ± 0.61
	CoDC	**93.04 ± 0.09**	**92.72 ± 0.06**	**91.39 ± 0.15**	**90.59 ± 0.05**
	Standard	89.02 ± 0.21	83.54 ± 0.21	76.37 ± 0.40	73.09 ± 0.27
	Co-teaching	93.08 ± 0.16	91.22 ± 0.24	88.49 ± 0.14	85.21 ± 0.28
	Co-teaching-plus	93.10 ± 0.13	89.57 ± 0.19	83.58 ± 0.43	73.28 ± 0.69
	JoCoR	95.01 ± 0.08	94.62 ± 0.08	92.65 ± 0.11	89.95 ± 0.07
SVHN-exp	Co-learning	91.67 ± 0.18	90.60 ± 0.23	86.52 ± 0.40	81.97 ± 0.58
	Co-LDL	95.33 ± 0.27	93.77 ± 0.35	89.58 ± 0.61	82.08 ± 1.56
	CoDis	92.80 ± 0.17	91.09 ± 0.19	86.89 ± 0.11	81.25 ± 0.13
	Bare	95.42 ± 0.14	92.51 ± 0.23	89.48 ± 0.33	83.31 ± 0.65
	CoDC	**95.68 ± 0.04**	**95.28 ± 0.07**	**93.97 ± 0.05**	**93.47 ± 0.10**
	Standard	88.90 ± 0.15	83.33 ± 0.20	76.92 ± 0.29	72.57 ± 0.24
	Co-teaching	92.92 ± 0.10	91.17 ± 0.10	88.33 ± 0.25	85.77 ± 0.26
	Co-teaching-plus	92.73 ± 0.13	89.41 ± 0.20	82.28 ± 0.37	73.94 ± 0.47
	JoCoR	95.34 ± 0.04	94.88 ± 0.07	93.56 ± 0.06	91.00 ± 0.07
SVHN-line	Co-learning	91.90 ± 0.09	90.70 ± 0.16	88.08 ± 0.25	81.80 ± 0.46
	Co-LDL	95.28 ± 0.12	93.40 ± 0.19	88.79 ± 0.25	83.66 ± 0.40
	CoDis	92.94 ± 0.17	90.78 ± 0.19	86.71 ± 0.11	80.93 ± 0.13
	Bare	95.40 ± 0.11	93.38 ± 0.22	89.62 ± 0.16	86.35 ± 0.72
	CoDC	**95.96 ± 0.06**	**95.77 ± 0.06**	**95.01 ± 0.06**	**93.56 ± 0.06**

**Table 3:** Test accuracy (%) on Clothing1M. The best results are in bold.

Method	Acc
Standard	67.22
Co-teaching	69.21
Co-teaching-plus	59.32
JoCoR	70.30
Co-learning	68.72
Co-LDL	71.10
CoDis	71.60
Bare	70.32
CoDC	**72.81**

**Table 4:** Top-1 and Top-5 test accuracy (%) on the WebVision1.0 and ILSVRC12 datasets. The best results are in bold.

	Dataset	WebVision		ILSVRC12	
Method		**top1**	**top5**	**top1**	**top5**
Co-teaching		63.58	85.20	61.48	84.70
Co-teaching-plus		68.56	86.64	65.60	86.60
JoCoR		61.84	83.72	59.16	84.16
Co-LDL		69.74	84.26	68.63	84.61
CoDis		70.52	87.88	66.88	87.20
Bare		69.60	88.84	66.48	88.76
CoDC		**76.96**	**91.56**	**73.44**	**92.08**

**Table 5:** Ablation study on CIFAR-100 under symmetric noise at a 40% noise rate.

Modules				Acc
$L_{\mathrm{select}}$	$L_{\mathrm{mlc}}$	$L_{\mathrm{balance}}$	$L_{\mathrm{pl}}$	
				33.20
✓				64.74
✓	✓			65.91
✓	✓	✓		68.38
✓	✓	✓	✓	68.76

**Table 6:** Comparison of running time between the proposed method and four co-teaching-based methods.

Method	Acc (%)	Time (h)
Co-teaching	77.16	1.34
Co-teaching-plus	71.49	1.62
Co-LDL	88.89	1.87
Bare	78.90	4.02
CoDC	90.15	1.76

## Data Availability

Data are contained within the article.
